# A Study of the Disputed Point in Local Anesthesia*Read before the Joint Meeting of the Interstate Association of Anesthetists and the National Dental Association at Louisville, Ky., July, 1916. Reprinted from *Dental Summary*.

**Published:** 1917-02

**Authors:** S. L. Silverman

**Affiliations:** Atlanta, GA.


### A STUDY OF THE DISPUTED POINT IN LOCAL ANESTHESIA. *

BY S. L. SILVERMAN, D.D.S., ATLANTA, GA.

*Professor of Local Anesthesia and Radiology; Associate Professor in Oral Surgery, Atlanta Dental College.*

In the discussion of this subject it is my aim to avoid predilections for, and prejudices to, any drug or method, permitting the drug or technic to stand on its own merits.

Much that I will say can only be verified or disproved in the clinic, the laboratory offering very little or no opportunity towards a satisfactory solution of certain phases of local anesthesia.

Some time ago Dr. Leo Stern, of New York, published an article [1] in which he maintained that infiltration anesthesia with novocain-suprarenin, resulted in hyperemia and death of pulps. In another and later publication [2] he elaborates on his first observations and reaches the conclusion that, ''infiltration anesthesia should *never* be used in the oral cavity.''

These are startling case records and necessarily result in a startling conclusion.

It would not be fair to myself if I did not here, at the outset, reiterate [3] that I, personally, rarely employ infiltration in my work. And my sole purpose is to learn whether or not it can be employed with safety.

In order to freshen your memory with the controversy that has arisen between Dr. Stern and myself, permit me to briefly quote the substance thereof:

I criticised [3] his advocating the pumping motion, ''moving the syringe backward and forward,'' when perform-

* Read before the Joint Meeting of the Interstate Association of Anesthetists and the National Dental Association at Louisville, Ky., July, 1916. Reprinted from *Dental Summary*.

ing the maxillary tuberosity and mandibular injections, because this method tunnelled through the tissue unnecessarily, and because[3] it would, by repeated jabs, do the things that those who practiced this method wished to avoid. To this, in answer to my criticism[4] he replies that Braun advocates this practice and then quotes Braun as follows:

"To avoid injecting large quantities of the anesthetic into a vein (and thus avoiding toxic effects) the syringe must be in constant motion, injecting during the insertion and withdrawal of the needle."

As is obvious from this quotation, Braun does not advocate pumping, and his fear of injecting directly into a vein is unwarranted, as applies to the dental regions; and, moreover, such accident is harmless, providing the solution is injected slowly. As a matter of information, I must add that it was Riethmuller who, in October, 1914, first called my attention to the adoption of the pumping motion, but has since concurred in my denunciation of the practice.

Let us now examine the most interesting part of the contention, that is, whether or not to employ infiltration.

That thousands of operators throughout the world employ infiltration anesthesia without the above-mentioned sequelæ, is a fact too obvious to mention. And I haven't either in print or correspondence been able to find other instances than those Dr. Stern mentions. Desiring, therefore, to test this point of contention in as near a laboratory manner as possible, I hit upon a plan that I think is certain to either commend or condemn it, but of this we shall speak later.

Dr. Stern is of the opinion that the "vaso-constrictor action of suprarenin," plus the "mechanical damage, subsequent to the great pressure required to force the solution into deep structure," are to blame for death of pulps; to which I must say that by diffusion and not by pressure, does the solution pass through the bone, and as Dzierzawsky has shown, using colored solutions in subperiosteal injections, it does not require long for the bone to be permeated, and, however delicate tissue may be, it still has recuperative powers and the action of suprarenin as will be shown later, appears to have no detrimental effect.

But these again are merely processes of laboratory reasoning. Permit me then to acquaint you with the experiments started:

Realizing that the average clinic is not a suitable place for the results sought, because after infiltration it is rather uncertain as to whether the patient will return at stated intervals for the purpose of ascertaining the vitality of pulps, I asked the first-year students of our college to volunteer for infiltration injections just for the experiment. I chose the first-year students for an obvious reason, namely, I have access to them for at least three years for observation.

The injections were all made in the presence of class members (whom on this occasion I wish to sincerely thank) and the technic employed was as follows:

The vitality of the right upper lateral was ascertained, and when found devitalized, the left upper lateral was chosen, and in no case did we have to leave these regions. The gum was iodinized, and one and one-half cc. of a two per cent. novocain-suprarenin solution was injected, and the area massaged. In no case has there been a report of trouble, and upon opening of college I shall at intervals re-examine our cases for this report. These experiments are necessarily slow, but we must build on facts.

A very important and probable factor where death of pulps has occurred following infiltration anesthesia, is the injury caused by over-heating with stone or bur. Though the tooth under anesthesia be insensitive to grinding, it should not be subjected to prolonged friction. A stream of water should be directed into the cavity during excavation.

Nor can the fact be entirely overlooked that some teeth we are called upon to treat are, as far as the pulps are concerned, already in a semi-devitalized state, and would without any sort of an injection, die.

We now come to a very interesting phase of my subject. What is the cause of partial collapse under novocain, the pallor, the trembling, etc.? Various authors predicate the causes, but I have never seen any one book or article that lists all of the causes that induce these results.

Thus Dr. Kurt Thoma is certainly in error when he imputes the tremor, pallor, etc,. to suprarenin. And the "T" tablet which he advocates will produce tremors similar to the "E" because it is the novocain, and not the suprarenin that is to blame. The solution that we usually employ, is made by dissolving one "E" tablet in one cc. of Ringer, the result being a two per cent. novocain solution, and the suprarenin in one to twenty thousand solution. Dr. Thoma would have suprarenin in one to seventy thousand, and therefore be of no practical value. When we realize that physicians daily use a one to one thousand solution (fifteen drops of same) with impunity, excepting in the aged, it stands as a safe procedure. *Moreover, the most practical test shows that pure novocain or cocain when applied to an open wound, causes this tremor to appear, yet when suprarenin one to one thousand is applied, this effect is not to be noticed.*

I have injected pure novocain into my forearm subcutaneously with the typical novocain tremor, which, by the way is preventable, but on injecting two cc. of suprarenin solution containing the same amount of the drug that is contained in two "E" tablets (viz.: .00010 of a gm.) this effect could not be produced. The reason for this is obvious: when novocain or any other local anesthetic is injected into the system, part of it immediately enters the circulation and as it passes the cardiac and respiratory brain centers, it affects them just sufficiently to create this flutter, this disturbance.

And now, can this novocain tremor be prevented? Or are we to allow a fear to spread throughout the profession and curtail its use? Already there is spreading a belief that there exists a novocain idiosyncrasy. And there is as much truth to that as there is to the superstition that there exists an idiosyncrasy to cocain. I hope that no one in this intelligent body is of that opinion, either as concerns novocain or cocain. Certainly no one here believes that when sloughing resulted after some careless pioneer injected a cocain solution that had stood so long on a dusty shelf, and had become so mouldy that it seethed with infection. I repeat, no one would credit the drug in this event, nor is it necessary to spend very much time ascertaining the truth of this statement, when you realize that in all the literature you never have heard of a clean cocain preparation ever having affected the most sensitive of tissue, viz.: the eye, the urethra. And while on the subject, let me say that were it not for the fact that cocain is habit-forming, and can not be sterilized by boiling, it would make an excellent substitute for novocain, in spite of its toxicity. But here you must be able to differentiate between toxicity as that term refers to the central nervous system, and local destruction of tissue (sloughing). The latter, cocain is incapable of doing, and its toxic effect on the central nervous system can also be prevented by proper care.

I suppose it is needless to state before this audience that in order to obviate tremors, partial faints, pallor and so forth, we must inject very, very slowly, and must have the patient in as near a supine position as is convenient.

The term "slow" is a relative one. Some operators empty a two cc. syringe in five seconds and think that it is a slow injection. Others double this time and think that it is an exceedingly slow injection. But if you would remember one thing of what I have said, please bear this in mind: *Take sixty seconds by the clock to empty your syringe, and you will have no experiences to relate.* Dosage matters but little; I have often used more than a tube of ''E'' tablets in surgical operations on the jaws. Concentration is of more moment than dosage, and even concentration is of less importance than the slowness of injection.

In this connection I wish to call your attention to the dangerous maxim promulgated by Seidel at the Muenster Congress where he remarks that ''It makes no difference whether a certain dose is injected in weak or strong concentration, as long as the total dose of .2 gm. is not transgressed.''[5] This certainly is dangerous teaching, and even the novice is aware that no local anesthetic is so harmless as to be used irrespective of concentration. A five grain tablet of bichlorid of mercury is certainly less toxic in a gallon of water than it would be in an ounce of the same. And so with .2 gm. of novocain.

Position has a great deal to do with prevention of the above-mentioned results, and the more erect the patient is sitting, the slower should the injection be executed.

When a patient is in a fainting condition, our first step is to place him in a supine position, so why not approach as near this position as is convenient and obviate chance? You often have heard the expression, ''he or she *fell* fainting,'' but I do not remember hearing of a faint from any cause while lying down. Attention to these details is productive of uneventful injections, and the so-called idiosyncrasies are not to be observed.

---

1 Local Anesthesia in Dentistry, September, 1915, *Items of Interest.*

2 January, 1916, *Dental Cosmos.*

3 See my letter in December, 1915, *Items of Interest.*

4 February, 1916, *Dental Items of Interest.*

5 Quoted from *Thoma's Oral Anesthesia.*

